# The Use of Coenzyme Q10 in Cardiovascular Diseases

**DOI:** 10.3390/antiox10050755

**Published:** 2021-05-10

**Authors:** Yoana Rabanal-Ruiz, Emilio Llanos-González, Francisco Javier Alcain

**Affiliations:** 1 Department of Medical Sciences, Faculty of Medicine, University of Castilla-La Mancha, 13071 Ciudad Real, Spain; Yoana.Rabanal@uclm.es (Y.R.-R.); Emilio.Llanos@uclm.es (E.L.-G.); 2 Oxidative Stress and Neurodegeneration Group, Regional Centre for Biomedical Research CRIB, University of Castilla-La Mancha, 13071 Ciudad Real, Spain

**Keywords:** Coenzyme Q10, ubiquinone, oxidative stress, heart failure, cardiac surgery, hypercholesterolemia, atherosclerosis, hypertension, endothelial dysfunction

## Abstract

CoQ10 is an endogenous antioxidant produced in all cells that plays an essential role in energy metabolism and antioxidant protection. CoQ10 distribution is not uniform among different organs, and the highest concentration is observed in the heart, though its levels decrease with age. Advanced age is the major risk factor for cardiovascular disease and endothelial dysfunction triggered by oxidative stress that impairs mitochondrial bioenergetic and reduces NO bioavailability, thus affecting vasodilatation. The rationale of the use of CoQ10 in cardiovascular diseases is that the loss of contractile function due to an energy depletion status in the mitochondria and reduced levels of NO for vasodilatation has been associated with low endogenous CoQ10 levels. Clinical evidence shows that CoQ10 supplementation for prolonged periods is safe, well-tolerated and significantly increases the concentration of CoQ10 in plasma up to 3–5 µg/mL. CoQ10 supplementation reduces oxidative stress and mortality from cardiovascular causes and improves clinical outcome in patients undergoing coronary artery bypass graft surgery, prevents the accumulation of oxLDL in arteries, decreases vascular stiffness and hypertension, improves endothelial dysfunction by reducing the source of ROS in the vascular system and increases the NO levels for vasodilation.

## 1. Introduction

According to the European Social Survey, the total prevalence of European people reporting heart or circulation problems in 2014, for all countries combined, was 9.2%. Within the top eight broad causes of death in elderly people, cardiovascular diseases (CVD) remain the most common worldwide (31.5%) [1], although other age-related and chronic diseases are diabetes (6%) and Alzheimer’s disease (3%), which are both closely related to cardiovascular diseases. In particular, diabetes causes peripheral vascular dysfunction and cardiovascular disease constitutes a risk factor for the development of Alzheimer’s disease [2].

Advanced age is the major risk factor for vascular disease since it leads to alterations in the structure and function of both the heart and the vascular wall, which are involved in the development of left ventricular hypertrophy and arterial stiffness, mitochondria dysfunction, increased oxygen species generation and abnormal calcium handling due to a reduction in calcium reuptake by the myocardial sarcoplasmic reticulum calcium adenosine triphosphatase (SERCA2a). Furthermore, age-associated increases in collagen deposition and decrease in elastin secretion cause arterial stiffness, which elevates systolic pressure, an independent risk factor for CVD [3]. Chronic exposure to elevated systolic pressure also leads to left ventricular hypertrophy. The second main feature of vascular aging is generalized endothelial dysfunction. Endothelial dysfunction reduces vasodilation by increasing proinflammatory cytokines and oxidative stress that reduces NO bioavailability and increases oxidized low-density lipoproteins (LDL), thus resulting ultimately in atherosclerosis (for reviews, see [3,4,5]). With age, oxidative stress increases in the human arterial system, and several lines of evidence link oxidative stress and mitochondrial dysfunction in CVD [6,7]. These observations have led to hypothesize that supplementation with coenzyme Q10 (CoQ10), an electron carrier in the mitochondrial respiratory chain, which is known to be a powerful antioxidant, could improve cellular bioenergetics in CVD [8,9]. This review summarizes the current evidence for the use of CoQ10 in the treatment of cardiovascular diseases such as heart failure, left ventricular thickening, cardiac surgery, hypertension, hypercholesterolemia, and endothelial dysfunction (Table 1), thus highlighting the recently described molecular mechanisms to explain the beneficial role for CoQ10 in the treatment of these diseases.

## 2. Physiology of CoQ10

CoQ10 links basic aspects of energy metabolism and antioxidant protection. This natural antioxidant plays a major role in cellular metabolism since it contributes to oxidative phosphorylation by mediating electron transfer between the Complexes I/II and the Complex III in the mitochondrial inner membrane but is also present in all cellular membranes and blood in both high-density lipoproteins (HDL) and LDL. The mitochondria act as an inhibitor of the mitochondrial permeability transition pore in the inner mitochondrial membrane, thereby inhibiting apoptosis [43].

Most of the oxygen taken up by human cells is reduced to water in the mitochondrial electron chain. Still, during these reactions, reactive oxygen species (ROS) such as superoxide (O_2_^•−^), H_2_O_2_ and hydroxyl radical (HO^•^) are generated [44]. CoQ10 exists in three alternate redox states: fully oxidized or ubiquinone (CoQ10); partially reduced, ubisemiquinone (CoQ10H), which is a free radical, and fully reduced or ubiquinol (CoQ10H_2_). CoQ10H_2_, a component of the mitochondrial respiratory chain [45], is well located in membranes close to the unsaturated lipid chains and has been established as a general antioxidant in membranes due to its activity as a primary scavenger of free radicals [44,46,47]. Moreover, CoQ10H_2_ is much more efficient in inhibiting LDL oxidation than other antioxidants such as β-carotene, or α-tocopherol [48]. CoQ10 can be recycled by four enzymes, NADH cytochrome *b*_5_ reductase, NQO1 oxidoreductase, NADPH coenzyme Q reductase, and the selenoprotein thioredoxin reductase [45,49]. CoQ10H_2_ can also regenerate other antioxidants such as α-tocopherol and ascorbate [50,51]. Furthermore, human promyelocytic HL-60 cells stabilize extracellular ascorbate in a CoQ10-dependent pathway, an important cellular function for the maintenance of the antioxidant system [50,52,53] that protects plasma lipids against detectable peroxidative damage induced by aqueous peroxyl radicals [47].

CoQ10 is endogenously produced and ubiquitous in all cells. Its synthesis requires several steps: the modification of the quinone (head group), the biosynthesis of isoprenoids (tail), attachment of head and tail, and the head group modifications. The precursor of the benzoquinone ring (head group) is 4-hydroxybenzoate, and the isoprenoid side chain starts from acetyl-CoA to generate farnesyl pyrophosphate through the mevalonate pathway. Then, 4-hydroxybenzoate and decaprenil pyrophosphate are condensed by the enzyme polyprenil 4-hydroxybenzoate transferase (encoded by COQ2), and finally, different enzymes (encoded by COQ3-8) catalyze methylation, decarboxylation and hydroxylation reactions to synthesize CoQ10 [54].

The highest amount of CoQ10 in cells is found in mitochondria, but CoQ10 distribution is not uniform among different organs, showing particularly high levels in the heart, kidney and liver [55]. During aging, CoQ10 concentration decreases in the pancreas, heart and plasma and shifts from the reduced to the oxidized redox status. This oxidation is associated with a loss of the antioxidant capacity that impacts tissue and lipoprotein protection in the blood [56,57,58].

Although CoQ10 is also found naturally in dietary sources, nutritional supplementation of CoQ10 does not increase tissue levels above normal [45]. CoQ10 absorption follows the same process as that of lipids in the gastrointestinal tract, but the uptake in the whole body ranges between 2 and 3% of the total dose. CoQ10 is reduced to ubiquinol either during or following absorption, and intestinal absorption is three-fold faster when CoQ10 is administered with food intake [55]. Ubiquinol shows superior bioavailability to ubiquinone (oxidized form) [59]. Because of its insolubility in water, a variety of formulations have been developed to solubilize CoQ10 and promote better absorption. The highest bioavailability has been found in nanoparticulated CoQ10, followed by solubilized, oil-emulsioned, and finally, powder [55].

## 3. CoQ10 and Heart Failure (HF)

The rationale of the use of CoQ in HF is that the loss of contractile function is due to an energy depletion status in the mitochondria that have been directly associated with low endogenous CoQ10 levels. Plasma and myocardial deficiency of CoQ10 have been demonstrated in endomyocardial biopsy samples from patients categorized according to the guidelines of the New York Heart Association (NYHA). These data revealed that myocardial deficiency of CoQ10 was linked to the severity of the disease, which was indeed reduced by therapy with oral administration of 90 mg of CoQ10 [10]. In another study, patients with severe hypertrophic cardiomyopathy that were treated with an average of 200 mg/day of CoQ10 improved in symptoms of fatigue and dyspnea with no side effects, and the mean interventricular septal thickness showed a 24% reduction [11]. Indeed, patients receiving oral CoQ10 (300 mg/day) for 2 weeks before cardiac surgery (n = 62) increased CoQ10 levels in serum (*p* < 0.01), atrial trabeculae (*p* < 0.001), and isolated mitochondria (*p* < 0.002) compared with that of patients receiving placebo (n = 59). Additionally, an increased efficiency of mitochondrial respiration (adenosine diphosphate/oxygen ratio) was observed (*p* < 0.12) [12]. This improvement in mitochondrial efficiency has also been demonstrated in a swine model of chronic myocardial ischemia, wherein CoQ10 supplementation (400 mg/day) for 4 weeks enhanced nuclear-bound PGC1-α, indicating the activation of mitochondrial biogenesis, and increased the expression of antioxidant proteins within the mitochondria [60].

Plasma CoQ10 concentration has been established as an independent predictor of mortality in chronic HF [61]. There have been numerous trials addressed to examine the effect of CoQ10 in improving HF symptoms during the past 30 years. Madmani et al. evaluated the randomized controlled trials that assessed the beneficial and harmful effects of CoQ10 in patients with HF until January 2013. The authors reported inconclusive results on the benefits or harms of coenzyme Q10 in heart failure since the existing data were derived from small and heterogeneous trials that focused on physiological measures. In addition, many of the included trials were not powered to address major clinical endpoints [62]. 

To prevent these limitations, Mortensen et al. conducted a randomized controlled multicenter trial addressed to evaluate CoQ10 as adjunctive treatment in chronic HF [13]. This study included primary short-term endpoints at 16 weeks (NYHA functional class, 6-min walk test, and N-terminal pro–B-type natriuretic peptide) and a primary long-term endpoint at 2 years (major adverse cardiovascular events consisting of unplanned hospital stay for worsening HF, cardiovascular death, mechanical assist implantation or urgent cardiac transplantation). The trial enrolled a total of 420 patients whose HF duration was approximately 3 years. After 16 weeks, serum CoQ10 levels significantly increased up to 3 times the baseline value in the CoQ10-treated group. Although no significant differences were found in heart rate, blood pressure or echocardiographic measurements at short-term endpoints, long-term endpoints were reached by 15% (n = 30) of the patients in the CoQ10 group versus 26% (n = 57) in the placebo group: cardiovascular mortality (n = 18, 9%) was lower compared to the placebo group (n = 34, 16%) corresponding to a relative reduction of 43%; all-cause mortality was lower in the CoQ10 group (n = 21, 10%) than the placebo group (n = 39, 18%), corresponding to a relative reduction of 42%.

Furthermore, the incidence of hospital stays due to worsening HF was significantly lower in the CoQ10 group (n = 17, 8%) versus the placebo group (n = 31, 14%). Finally, a significant improvement in NYHA class was observed in the CoQ10 group after 2 years. The beneficial effects of CoQ10 supplementation were in addition to those provided by beta-blockers and angiotensin-converting enzyme inhibitors or angiotensin receptor blockers [13].

Similarly, a double-blind study with nonischemic HF patients (72 men and 30 women, aged 62.3 years) demonstrated that the use of 30 mg/day as adjuvant treatment attenuated the incidence of atrial fibrillation (3 patients (6.3%) in the CoQ10 group and 12 patients (22.2%) in the control group, *p* = 0.02) [14].

Geographical differences in patient characteristics and management can influence the outcomes in HF trials. However, the therapeutic efficacy of CoQ10 shown in the above-mentioned Q-SYMBIO study was confirmed in a subsequent trial, which included a European sub-population of 231 patients. The results obtained from this group revealed that CoQ supplementation reduced all-cause mortality, cardiovascular mortality, hospitalization, and improved symptoms [15].

These conclusions were in line with previous results published by Alehagen et al. in a 5-year prospective randomized, double-blind placebo-controlled trial among 443 healthy elderly Swedish citizens aged 70 to 88 treated with a combined supplementation of selenium and CoQ10 or a placebo. In this study, cardiovascular mortality reached 12.6% in the placebo group and 5.9% in the active treatment group, thus showing a significant reduction (HR, 0.45 [95% CI 0.24–0.89]; *p* = 0.02). Additionally, all-cause mortality was reduced in the active treatment group vs. the placebo group (12.7% vs. 16.2%, respectively). A significant difference in N-terminal pro–B-type natriuretic peptide plasma concentration levels between the two groups was found at 24 months (*p* = 0.048) which was further pronounced at 48 months (*p* = 0.014). Furthermore, the cardiac function score was found to be significantly better, according to echocardiography, in the active supplementation group compared to the placebo group [16]. All of these protective actions of CoQ10 supplementation were not restricted to the intervention period but persisted during the 10-year follow-up period [17]. It is important to note that supplementation was cardio-protective exclusively in those participants with a low selenium concentration <85 µg/L, but not in those whose serum selenium levels were >85 µg/L at inclusion [18]. This intervention also reduced fructosamine levels, which are directly associated with cardiovascular risk [19]. The rationale to combine selenium with CoQ10 is based on the need for the selenium-dependent thioredoxin reductase to reduce CoQ10 to the active form ubiquinol, and a deficiency of selenium could therefore restrict the cells ability to obtain the optimal concentrations of ubiquinol [49].

As patients age, mitochondrial ROS production significantly increases in the myocardium, escalating the risk of myocardial oxidative injury and coronary stiffens due to increased collagen deposition [63,64,65]. A randomized, double-blind, placebo-controlled trial addressed to explore whether CoQ10 supplementation improved endothelial function in patients with ischemic left ventricular systolic dysfunction (LVSD, left ventricular ejection fraction <45%) [20], studied the effects of CoQ10 supplement (300 mg/day, n = 28) vs. placebo (controls, n = 28) for 2 months on brachial flow-mediated dilation (FMD). After 8 weeks, CoQ10 in plasma increased from 1.08 to 3.24 µg/mL in the treatment group, while no significant differences were observed in the placebo group (from 0.95 to 0.98 µg/mL). The absolute increase in plasma CoQ10 levels significantly correlated with an improvement in brachial FMD.

Indeed, the beneficial effect of CoQ10 supplementation was more pronounced in those patients exhibiting more severe endothelial dysfunction and more severe mitochondrial dysfunction (measured as lactate/pyruvate ratio in plasma). Furthermore, the reduction in plasma lactate/pyruvate ratio significantly correlated with an improvement in FMD, indicating that the improvement in endothelial function was directly associated with an improvement in mitochondrial function [20]. It has also been reported that CoQ10 treatment increased elastin gene expression in cultured skin fibroblasts [66] and supplementation with both, CoQ10 and selenium, also reduced biomarkers of fibrogenic activity (cathepsin S, endostatin, galectin 3, growth differentiation factor-15, matrix metalloproteinases 1 and 9, and tissue inhibitor of metalloproteinases 1) suggesting that the improvement in cardiac function is preceded by the reduction of fibrosis, thus explaining some of the positive clinical effects triggered by the intervention [21]. Moreover, CoQ10 supplement at a dose of 150 mg has been shown to decrease oxidative stress and increase antioxidant enzyme activities in patients with coronary artery disease (CAD), although its correlation with clinical benefits still needs to be elucidated [67].

Multiple sources of evidence indicate that the renin–angiotensin–aldosterone system plays a significant role in developing ventricular hypertrophy. Specifically, angiotensin II generates superoxide in mice hearts in vivo, which activates both c-Jun N-terminal kinase (JNK) and p-38 mitogen-activated protein kinase (MAPK) via apoptosis signal-regulating kinase-1 (ASK1). After ASK1 activation, cardiac hypertrophy, apoptosis, fibrosis, and coronary arterial remodeling occurred in the wild-type mice. This effect was significantly attenuated in ASK1^−/−^ mice or with the antioxidant tempol [65]. The role of CoQ10 as an efficient antioxidant to improve angiotensin II-induced oxidative stress in human endothelial cells has been demonstrated [68]. Furthermore, CoQ10 supplementation of the offspring postnatal diet protects against premature cardiovascular aging in a rat by rectifying the cardiac cellular stress, antioxidant defense alterations, telomere shortening, cellular senescence and apoptosis [69].

The increased collagen deposition that occurs during aging also contributes to left-ventricular thickness. CoQ10 reduced the systemic lipid peroxidation and ventricular superoxide and limited diabetes-induced cardiomyocyte hypertrophy, apoptosis and cardiac fibrosis in *db/db* mice preserving diastolic function and SERCA2a expression [64]. Furthermore, in the 3×Tg-AD mice, a model for Alzheimer’s disease, supplement of ubiquinol and ascorbate prevented the collagen deposition in the cerebrovascular basement membrane [70].

Arterial stiffness is an independent risk factor for cardiovascular diseases [71]. A study matched by gender in patients with heterozygous familial hypercholesterolemia (FH) reported a negative association between plasma CoQ10 and arterial stiffness. FH patients treated long-term with high dose statin therapy, an inhibitor of HMG-CoA reductase and CoQ10 biosynthesis, exhibited higher arterial stiffness than untreated controls with similar lipid levels [72].

The positive effect of CoQ10 on preserving arterial elasticity was evaluated in a placebo-controlled, double-blinded randomized trial that enrolled 65 Los-Angeles County firefighters who were randomized to receive four tablets of aged garlic extract (AGE, 300 mg/tablet) plus CoQ10 (30 mg/tablet) or placebo. After an adjustment for cardiovascular risk factors and statin therapy, the vascular stiffness, measured as pulse-wave velocity, showed a mean decrease of 1.21 m/s in the AGE/CoQ10 compared with the placebo group (*p* < 0.005). Similarly, endothelial function also improved significantly in the treated group [22]. Furthermore, a double-blind, placebo-controlled, randomized clinical trial carried out on 40 non-smokers moderately hypercholesterolemic subjects revealed that arterial stiffness significantly improved after 10 mg monacolin plus 30 mg CoQ10 treatment [pulse wave velocity (PWV) after treatment: −4.7%; PWV after placebo: +1.1%; *p* < 0.05] [23].

Nonetheless, it has also been reported that supplementing with 200 mg/day of CoQ10 for 12 weeks to obese patients does not significantly affect the brachial–ankle pulse wave velocity, an indicator of arterial stiffness [24].

## 4. CoQ10 in Cardiac Surgery and Coronary Arterial Disease (CAD)

Oxygen free radicals generated during and after tissue ischemia and reperfusion constitute a causative factor for the structural and metabolic damage during heart surgery. In 1993, Judy et al. examined the effects of CoQ10 therapy on cardiac function and the clinical recovery course in high-risk patients during heart surgery. These patients presented blood CoQ10 deficiency, but the presurgical CoQ10 treatment (100 mg/day) significantly (*p* < 0.01) improved blood, myocardial CoQ10 and myocardial ATP, cardiac pumping and left ventricular ejection fraction compared to the control group [25]. Additionally, a relation between low plasma CoQ10 concentration and CAD has been reported [73].

Supplementing with 300 mg/day of CoQ10, among other antioxidants, to reduce these stresses prior to cardiac surgery group showed a significant improvement from preoperative baseline in the quality-of-life questionnaire scores [26]. Furthermore, preoperative oral CoQ10 therapy (300 mg/day for 2 weeks) in patients undergoing elective cardiac surgery (n = 62), significantly increased CoQ10 levels in serum, atrial trabeculae and isolated mitochondria compared with patients receiving placebo (n = 59), hence increasing myocardial tolerance to in vitro hypoxia-reoxygenation stress [12]. Moreover, oral CoQ10 therapy (150 to 180 mg/day) for 7 to 10 days preoperatively could improve clinical outcome in patients undergoing coronary artery bypass graft surgery since significantly fewer reperfusion arrhythmias, lower total inotropic requirement, mediastinal drainage, blood product requirement, and shorter hospital stays were observed when compared with the control group [27]. Pepe et al. summarized the nine controlled trials that had been published from 1982 to 2004 evaluating CoQ10 in cardiac surgery. All showed a beneficial effect except one [74]. In this particular study, patients were pretreated with 300 mg CoQ10 only 12 h before the procedure and a reduction of the preprocedural myocardial injury following elective percutaneous coronary intervention was not observed, although the hs-C reactive protein significantly decreased [28]. This lack of effectiveness in the treatment with CoQ10 may be due to an insufficient increase in the plasma levels of CoQ10 since plasma ubiquinol concentration had nearly reached a steady state by 2 weeks after the beginning of the treatment [55,74]. 

However, a meta-analysis focused on the prophylactic treatment with CoQ10 in patients undergoing cardiac surgery with cardiopulmonary bypass showed that CoQ10 administration before surgery significantly reduces the risk of requiring inotropic drugs after surgery in 53%, as well as the incidence of ventricular arrhythmias, although no significant differences were found in atrial fibrillation, cardiac index (measured in l/m^2^/min 24 h after surgery) and hospital stay. Since no adverse effects associated with CoQ10 administration were reported in any of the clinical trials, CoQ10 was determined to be safe even at high doses [29].

It has also been shown that circulating CoQ10 levels gradually decrease with time in patients with acute ST segment elevation myocardial infarction (STEMI), but higher plasma CoQ10 concentrations 1 month after primary angioplasty were associated with favorable LV remodeling and systolic function 6 months after STEMI [75]. A preoperative biomarker for improving risk-stratification that has been incorporated into the Canadian guidelines is the N-terminal prohormone BNP (NT-proBNP). NT-proBNP levels predict adverse post-vascular surgery events. It has been demonstrated that preoperative administration of CoQ10 for 3 days prior to the elective vascular surgical procedure lowers perioperative NT-proBNP levels [30,76].

In summary, oral CoQ10 therapy (200 or 300 mg/day) for 7–14 days preoperatively could improve clinical outcomes in patients undergoing coronary and cardiac surgeries by improving mitochondrial respiration and increasing myocardial tolerance to oxidative stress.

## 5. CoQ10 Hypercholesterolemia and Atherosclerosis

Hypercholesterolemia refers to an increase in the normal levels of cholesterol in blood that predisposes to the development of cardiovascular diseases due to the deposit of atheroma plaques in the arteries. Statins were recommended as first-line therapy for the atherosclerotic cardiovascular disease since they constitute 3-hydroxy-3-methylglutaryl CoA reductase (HMG-CoA reductase) inhibitors, thereby reducing the synthesis of endogenous cholesterol and, consequently, LDL-cholesterol levels in plasma [77]. Statins inhibit the production of mevalonate, a precursor of both cholesterol and CoQ10, and statin therapy may lower plasma CoQ10 concentration also due to a reduction in LDLs levels [55]. Banach et al. (2015) evaluated the impact of statin therapy on plasma CoQ10 concentration through a meta-analysis and found a significant reduction following treatment with statins [78]. These results were confirmed by a recent meta-analysis, although the decrease of circulating CoQ10 was not closely associated with the duration of statin treatment [79].

There have been few clinical problems with the use of statins, and myopathies are considered the most serious, ranging from mild myalgia to fatal rhabdomyolysis [80].

CoQ10 supplementation in patients receiving statin therapy to relieve muscle pain has yielded inconclusive results. In a meta-analysis of randomized controlled trials on the effects of CoQ10 on statin-induced myopathy, the authors did not find any significant benefit of CoQ10 supplementation [31]. Conversely, the meta-analysis performed by Qu et al. concluded that CoQ10 supplementation ameliorated statin-associated muscle symptoms, supporting that CoQ10 could constitute a complementary approach to manage statin-induced myopathy. However, no reduction in plasma creatine kinase levels was observed after CoQ10 supplementation [32]. In accordance with this study, Derosa et al. reported that the addition of CoQ10 with half dosage statin in patients with preceding intolerance to statins improves the perception of myalgia, asthenia or pain. However, they found that CoQ10 plasma concentrations inversely correlated with creatine kinase levels [33].

Atherosclerosis is a chronic, generalized, and progressive disease that mainly affects medium-sized arteries. The main cause of atherosclerosis is hypercholesterolemia but also hypertension and hyperglycemia due to insulin resistance or diabetes [81]. Clinically, atherosclerosis manifests as ischemic heart disease, cerebrovascular disease, or peripheral arterial disease. The first stage consists of the internalization of cholesterol via circulating LDL in the arterial intima, which promotes endothelial activation/dysfunction [82]. An accumulation of LDLs in the arterial intima, wherein they may be modified by oxidation and aggregation, can be a sufficient cause of atherosclerosis. LDLs induce endothelial cells and smooth muscle cells (SMCs) to express adhesion molecules such as vascular cell adhesion molecule–1 (VCAM-1) and intercellular adhesion molecule–1 (ICAM-1) that interact with receptors on monocytes and stimulate their migration to the arterial intima [81].

Ubiquinol acts as a potent inhibitor of LDL lipoperoxidation in vitro when the lipoprotein is exposed to oxidants produced by activated human polymorphonuclear leukocytes [48], and it also constitutes the first lipid-soluble antioxidant consumed when isolated LDL or human plasma is exposed to a vast array of oxidants [83]. Furthermore, dietary CoQ10 has shown an anti-atherogenic effect by preventing the accumulation of aortic lipid peroxides in hypercholesteremic ApoE knockout mice [84]. In addition, CoQ10 suppressed oxidized low-density lipoprotein-induced macrophage foam cell formation by reversing cholesterol transport in macrophages, thereby inhibiting the progression of atherosclerosis. This specific mechanism reduces miR-378 expression and enhances the ATP-binding cassette transporter G1–mediated macrophage cholesterol efflux to high-density lipoprotein in ApoE knockout mice [85].

Additionally, changes in CoQ10 serum concentration positively correlated with cholesterol efflux to high-density lipoproteins (HDL) in human monocyte-derived macrophages from healthy individuals who received 100 mg CoQ10 twice daily for 1 week [86]. Finally, supplementation with CoQ10 plus AGE reduced the progression of coronary artery calcium, which can be used to track the progression of atherosclerosis, over a 1 year follow-up period and showed beneficial effects on vascular elasticity [22,34].

## 6. CoQ10, Endothelial Dysfunction and Hypertension

Endothelial dysfunction is driven primarily by nitric oxide (NO) deficiency and increased bioavailability of oxidizing ROS. NO displays a variety of functions within the vascular endothelium, such as vascular tone regulation or smooth muscle growth. It also reduces platelet aggression and adhesion, inhibits the interaction between leukocytes and the vessel wall, and neutralizes the oxidation of LDL cholesterol [87,88]. NO generation is catalyzed by the endothelial nitric oxide synthase (eNOS), which converts L-arginine to L-citrulline to reduce oxygen to form NO. This reaction is cofactored by tetrahydrobipterin (BH4) in the presence of flavin mononucleotide (FMN), flavin adenine dinucleotide (FAD) and nicotinamide adenine dinucleotide phosphate (NADPH) [89,90,91,92]. A shift in the equilibrium that favors NO deficiency and ROS formation leads to endothelial dysfunction, which has been directly linked to various diseases, including atherosclerosis, diabetes mellitus, CAD, hypertension, and hypercholesterolemia [93]. Indeed, increased oxidative stress is considered a major mechanism involved in the pathogenesis of endothelial dysfunction. The main sources of oxidative stress in the vasculature are xanthine oxidase (XO) in the bloodstream, NADPH oxidase (NOX) on the surface of endothelial cells, and the mitochondria [94]. The XO enzyme is produced in the liver and secreted into the blood. XO plays a key role in purine degradation and catalyzes the oxidation of hypoxanthine to xanthine and, subsequently, of xanthine to uric acid. As a byproduct of the XO-mediated degradation of purines, ROS (including O_2_^•−^ and H_2_O_2_) are generated [95]. XO binds to the endothelium, and their XO-derived O_2_^•−^ rapidly reacts with NO to generate ONOO^–^ [96]. 

Plasma XO activity has been established as an independent predictor of CVD and chronic kidney disease [96] and CoQ10 supplementation was shown to prevent the effects on XO expression in a rat model on cardiac aging based on nutritional programming [69]. In healthy humans, the plasma XO activity significantly increases with age from 38 to 65 years old [97]. Yet, a dietary intervention with selenium (200 µg/day) and CoQ10 (200 µg/day) in an elderly population showed a downregulation of the XO pathway, thus decreasing the levels of uric acid in plasma, which might indicate a reduced need for antioxidants because ROS were also lowered [35]. NOXs are a primary source of ROS in the vascular system and play a central role in cardiovascular health and disease. Among other vascular pathologies, NOXs are implicated in endothelial dysfunction [87]. Different NOXs have been identified in vascular smooth muscle (NOX1, NOX2 and NOX4), endothelium (NOX2 and NOX4), cardiomyocytes (NOX2 and NOX4) and vascular adventitium (NOX4, NOX2 and NOX1). All members of the family are membrane-bound enzymes that transfer electrons from NADPH to molecular oxygen, thereby generating O_2_^•−^ [98].

The major source of ROS is believed to be the NOX2 isozyme [99]. NOX2 activation produces O_2_^•−^, which reacts with NO to generate ONOO^−^, thus eliminating the NO available for vasodilation. ONOO^−^ and ROS oxidize the NOS cofactor BH4, which results in NOS uncoupling and ongoing production of ROS and damage to lipids, proteins, and DNA [94]. Indeed, ONOO^−^ is a crucial mediator of lipid peroxidation and protein nitration, including LDL oxidation which is critical for atherogenesis, and might cause apoptosis of endothelium or cells of the fibrous cap of unstable plaques [100]. These have major implications for endothelial dysfunction in the microcirculation and contribute to atherosclerosis, hypertension, congestive heart failure and ischemia-reperfusion injury. NOX2 seems to be primarily present in the endothelium and is responsible for endothelial dysfunction under disease conditions [87]. Even in unstimulated endothelial cells, NOX2 is constitutively active at a low level, contributing to the maintenance of vascular tone, but can be further stimulated acutely by agonists such as angiotensin II, oxLDL and a high glucose concentration [101,102,103]. 

CoQ10 supplementation has been shown to protect cultured endothelial cells against these insults by attenuating oxLDL, angiotensin II, glucose and β-peptide-induced ROS generation and reducing endothelial cell death [68,104,105,106,107,108]. Specifically, CoQ10 supplementation prevented the oxLDL-induced endothelial dysfunction through activation of AMPK that upregulates the Akt/eNOS/NO pathway and suppressed the PKC-mediated activation of NOX in human umbilical vein endothelial cells (HUVECs). Furthermore, CoQ10 also attenuated the oxLDL-mediated down-regulation of eNOS and the secretion of endothelin 1 (ET-1). It suppressed oxLDL-activated NF-κB and downstream inflammatory mediators, including expression of adhesion molecules, the release of proinflammatory cytokines (ICAM-1, VCAM-1, IL-6, TNF-α and NLRP3) and the adherence of monocytic THP-1 cells [107,108,109]. The preventive effects of CoQ10 against atherosclerosis might be achieved by improving mitochondrial function and promoting energy metabolism through the AMPK-YAP-OPA1 pathway [109].

Angiotensin II-induced oxidative stress and endothelial dysfunction is profoundly implicated in the pathogenesis of hypertension. Treatment of HUVEC with CoQ10 prevented the increase in expression levels of NOX2 and the upregulation of ICAM-1 and VCAM-1 [68].

In a recent study designed to evaluate the effect of metformin alone or in combination with CoQ10 on inflammatory changes in patients with type-2 diabetes mellitus (T2DM), CoQ10 add-on metformin therapy significantly declined the VCAM-1 and E-selectin serum levels [36]. In vivo, CoQ10 exclusively impacts on endothelium-dependent vasodilation since the relaxant responses of the rat aorta to acetylcholine were markedly potentiated after pre-incubation with CoQ10 or L-arginine, the substrate of eNOS [110]. In aged rats, the impaired vasodilation of noradrenaline-precontracted rings to acetylcholine was also improved upon CoQ10 supplementation, and the mechanism may involve augmented endothelial production of PGI_2_ [111]. Additionally, oral administration of CoQ10 attenuated high salt-induced hypertension in adult male Sprague-Dawley rats by reducing NOXs activity in the hypothalamic paraventricular nucleus [112].

In a multicentre, randomized, open-label, post-marketing clinical trial that enrolled 104 subjects, 52 subjects were treated with a once-daily oral formulation of a nutraceutical compound containing red yeast rice (rich in monacolin) and CoQ10, which was included to their diet for 2 months, and were compared with the 52 subjects following a diet program. A greater reduction of systolic blood pressure (−5.2 vs. −3.0 mmHg), diastolic blood pressure (−4.9 vs. 2.9 mmHg), total cholesterol (−17.2%), LDLC (−21.8%), triglycerides (−16.0%), and serum glucose (−3.4%) was observed in the treatment group compared to the control (*p* < 0.001 for all) [37]. Likewise, in the Al-Kuraishy et al. study, CoQ10 add-on metformin therapy showed a significant reduction in blood pressure changes and a significant increment in insulin sensitivity in patients with T2DM compared with baseline [36]. 

Another source of NOX activation is hyperglycemia. In high glucose-stimulated endothelial progenitor cells (EPC), proliferation, migration, Akt/eNOS activity and NO production were downregulated, and the administration of CoQ10 ameliorated these dysfunctions by upregulating eNOS and hemoxigenase 1 (HO-1) through the AMPK pathway. Furthermore, transplantation of CoQ10-treated EPCs under high-glucose conditions into ischemic hindlimbs improved blood flow recovery [106].

Nevertheless, the effect of CoQ10 on insulin sensitivity is contradictory because it has also been reported that 2 × 200 mg/day of CoQ10 supplementation for 8 weeks did not alter insulin secretory capacity and neither improved peripheral insulin sensitivity in statin-treated men and women [38]. On the contrary, T2DM patients supplemented with liquid ubiquinol (100 mg/day) had a slightly lower level of fasting glucose than the placebo group at week 4 and reduced HbA1c levels [39]. Moreover, whereas the insulin sensitizer agent metformin did not show a significant effect on LDL serum levels in T2DM patients, the combination of metformin and CoQ10 led to an amelioration of lipid profile and glucose indices, through the reduction of fasting blood glucose, fasting serum insulin and HbA1c along with a significant increment in the insulin sensitivity compared with the baseline [36].

A significant change in metabolic profiles after intervention with selenium (200 µg/day) and CoQ10 (200 mg/day) in an elderly population over four years has also been reported. Patients who received selenium plus CoQ10 showed a decrease in the polyunsaturated fatty acids arachidonic acid, eicosapenatenoic and docosahexaenoic acid, and an increase in saturated fatty acids (hexadecenoic acid, myristic and stearic acid) and monounsaturated fatty acids (hexadecenoic acid and oleic acid), suggesting that the intervention might be cardioprotective and has a positive effect on individuals with metabolic syndrome and/or T2DM. Likewise, the levels of essential amino acids, which are associated with T2DM, decrease with selenium and CoQ10 supplementation [35].

In 2015, Yang et al. reviewed the clinical trials addressed to evaluate CoQ10 treatment in chronic HF, hypertension and endothelial dysfunction and reported that CoQ10 had a remarkably anti-hypertensive effect and showed an improvement in the endothelial function in patients with CAD, HF and diabetes mellitus. However, in healthy humans, CoQ10 has no direct vasodilatory or hypotensive effect, suggesting a specific hypotensive effect of CoQ10 under the increased oxidative stress that occurs in hypertensive patients [9]. A recent randomized, double-blind placebo-controlled crossover pilot study showed that 400 mg/day of ubiquinol for 3 months leads to significant improvement in peripheral endothelial function in patients with HF with reduced ejection fraction [40].

FMD of the brachial artery is the most widely used non-invasive technique to evaluate endothelium-dependent and -independent vasodilation. A very recent systematic review and meta-analysis of 12 randomized controlled trials enrolling a total of 650 patients examined the effect of CoQ10 on metabolic and CVD-related risk profiles in individuals with diabetes or metabolic syndrome. This study concluded that CoQ10 supplementation showed greater potential to lower CVD risk in diabetic patients by lowering total cholesterol and LDL levels when compared to those on placebo, which was associated with an improvement of endothelial health [41].

The effect of ubiquinol in ameliorating dyslipidemia-related endothelial dysfunction, determined by FMD, was confirmed in another independent randomized clinical trial, which concluded that this effect was strongly related to enhancing NO bioavailability and partly mediated by increased LDL antioxidant protection [42].

## 7. Conclusions

**Figure 1:**
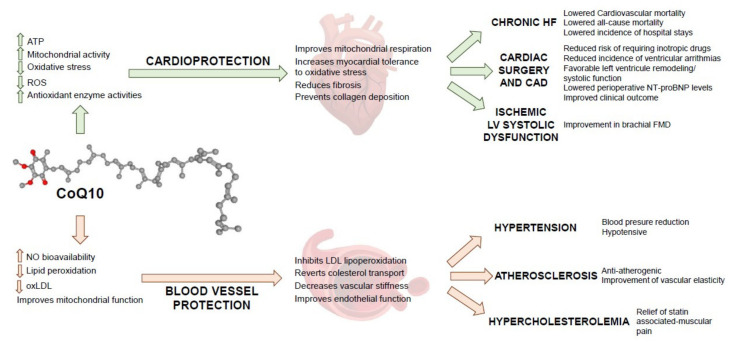
Mechanisms of action of CoQ10 in cardiovascular disease. ATP: adenosine triphosphate; ROS: reactive oxygen species; NO: nitric oxide; LDL: low-density lipoprotein; NT-proBNP: N-terminal prohormone BNP; FMD: flow-mediated dilation; CAD: coronary artery disease.

Figure 1 summarizes the potential mechanistic, physiological, and clinical benefits of CoQ10. Clinical evidence shows that CoQ10 supplementation with doses of 200 mg/day or higher for prolonged periods is safe, well-tolerated and significantly increases the concentration of CoQ10 in plasma, and reduces oxidative stress and mortality from cardiovascular causes. CoQ10 supplementation improves HF symptomatology and clinical outcome in patients undergoing coronary artery bypass graft surgery by enhancing mitochondrial respiration and increasing myocardial tolerance to oxidative stress. Dietary CoQ10 also modulates a number of risk factors through an anti-atherogenic effect that prevents the accumulation of oxLDL in arteries, decreases vascular stiffness and hypertension, improves endothelial dysfunction (by reducing the source of ROS in the vascular system) and increases NO levels for vasodilation.

## Figures and Tables

**Table 1:** Clinical trials and meta-analyses of CoQ10 in heart failure (HF), coronary artery disease (CAD), hypercholesterolemia, atherosclerosis, endothelial dysfunction and hypertension. N/A: not applicable.

	Reference Number	Author	Study Design	Number of Participants	Inclusion Criteria/Diagnosis	CoQ Daily Oral Dose	Intervention Period	Finding
**Heart failure (HF)**	[10]	Folkers (1985)	Clinical trial	43	Cardiomyopathy	90 mg	2–8 months	Profound increase both in cardiac function and in the quality of life of a failing cardiac patient.
	[11]	Langsjoen (1997)	Clinical trial	7	Severe hypertrophic cardiomyopathy	200 mg	3 or more months	Improvement in symptoms of fatigue and dyspnea. Reduction of the mean interventricular septal thickness.
	[12]	Rosenfeldt (2005)	Clinical trial	121	Patients undergoing elective cardiac surgery	300 mg	2 weeks before surgery	Improvement in mitochondrial function. Increased myocardial tolerance to in vitro hypoxia-reoxygenation stress.
	[13]	Mortensen (2014)	Randomized double-blind	420	Chronic HF	300 mg	16 weeks–2 years	Improvement of symptoms. Reduced major adverse cardiovascular events.
	[14]	Zhao (2015)	Double-blind	102	Nonischemic HF	30 mg	6–12 months	Attenuation of atrial fibrillation incidence.
	[15]	Mortensen (2019)	Randomized double-blind	231 European	Chronic HF	300 mg	3 months–2 years	Reduction of all-cause mortality, cardiovascular mortality, hospitalization and improvement of symptoms.
	[16]	Alehagen (2013)	Randomized double-blind placebo-controlled	443	Elderly Swedish citizens	200 mg and Selenium (200 μg)	5 years	Reduction of cardiovascular mortality.
	[17]	Alehagen (2015)	Prospective Randomized Double-Blind Placebo-Controlled trial	443	Elderly Swedish citizens	200 mg and Selenium (200 μg)	5 years	Significant reduction of cardiovascular mortality.
	[18]	Alehagen (2016)	Randomized Clinical Trial	668	Elderly Swedish citizens	200 mg and Selenium (200 μg)	4 years	Cardioprotection in those praticipants with a low selenium concentration.
	[19]	Alehagen (2020)	Prospective, randomized, double-blind placebo-controlled trial	219	Elderly community-living participants	200 mg and Selenium (200 μg)	6 and 42 months	Lower concentration of fructosamine Lower cardiac mortality. Less inflammation.
	[20]	Dai (2011)	Randomized, double-blind, placebo-controlled trial	56	Ischaemic LVSD (left ventricular ejection fraction <45%)	300 mg	8 weeks	Improvement in mitochondrial function and flow-mediated dilation (FMD).
	[21]	Alehagen (2018)	Clinical trial	443	Healthy elderly persons	200 mg and Selenium (200 μg)	4 years	Reduction of fibrosis. Improvement in cardiac function.
	[22]	Larijani (2013)	Double- blind randomized Clinical Trial	65	Los-Angeles County firefighters	30 mg and aged garlic extract (300 mg)	1 year	Improvement on vascular elasticity and endothelial function.
	[23]	Cicero (2016)	Double blind, placebo-controlled, randomized clinical trial	40	Moderately hypercholesterolemic	30 mg and monacolins (10 mg)	6 months	Improvement in LDL-cholesterolemia and arterial stiffness.
	[24]	Lee (2011)	Double-blind randomized controlled study	51	Obese	200 mg	12 weeks	No effect on arterial stiffness, fatigue index, metabolic parameters or inflammatory markers.
**Coronary artery disease (CAD)**	[25]	Judy (1993)	Case-control study (patients during heart surgery compared to placebo controls)	20	High-risk patients undergoing cardiac surgery	100 mg	14 days before and 30 days after surgery	Improvement in cardiac pumping and left ventricular ejection.
	[26]	Hadj (2006)	Clinical trial	16	Cardiac surgery patients	300 mg [and alphalipoic acid (300 mg), magnesium orotate (1200 mg), and omega 3 fatty acids (3 g)]	36 ± 7 days up until the day of operation	Lower systolic blood pressure. Reduction in levels of oxidative stress. Enhanced post-operative recovery.
	[27]	Makhija (2008)	Prospective, randomized, single-center clinical study	30	Patients scheduled for elective coronary artery bypass graft surgery	150 to 180 mg	7 to 10 days preoperatively	Fewer reperfusion arrhythmias, lower total inotropic requirement, mediastinal drainage, blood product requirement, and shorter hospital stays.
	[28]	Aslanabadi (2016)	Randomized Clinical Trial	100	Patients scheduled for elective percutaneous coronary intervention (PCI)	300 mg	12 h before procedure	No reduction of periprocedural myocardial injury following elective PCI. Decrease in hs-C reactive protein.
	[29]	de Frutos (2016)	Meta-analysis	327	Cardiac surgery requiring cardiopulmonary bypass	30–600 mg	from 12 h to 14 days before surgery	Reduced risk of requiring inotropic drugs after surgery. Lower incidence of ventricular arrhytrmias.
	[30]	Khan (2020)	Double-blind, randomized controlled trial	123	Vascular surgery	400 mg	3 days before vascular surgery	Lower perioperative NT-proBNP levels.
**Hypercholeterolemia and atherosclerosis**	[31]	Banach (2015)	Meta-analysis of randomized controlled trials	302	Patients receiving statin therapy	100–400 mg	From 30 days to 3 months	No benefit of CoQ10 supplementation in improving statin-induced myopathy.
	[32]	Qu (2018)	Meta-analysis of randomized controlled trials	575	Dyslipidemia/patients treated with statins	N/A	From 30 days to 3 months	Ameliorated statin-associated muscle symptoms.
	[33]	Derosa (2019)	Double-blind, randomized, placebo-controlled study	60 Caucasian	Dyslipidemia (intolerant to statins)	100 mg	3 months	Improvement in the perception of asthenia, myalgia or pain.
	[34]	Zeb (2012)	Placebo-controlled, double-blind, randomized trial	65	Intermediate risk firefighters	120 mg and aged garlic extract (1200 mg)	1 year	Beneficial effects on vascular elasticity. Reduced progression of coronary atherosclerosis.
**Endothelial dysfunction and hypertension**	[35]	Alehagen (2019)	Double-blind, randomised placebo-controlled prospective study	443	Healthy elderly population	200 mg and Selenium (200 μg)	4 years	Significant changes in the pentose phosphate, the mevalonate, the betaoxidation and the xanthine oxidase pathways. Changes in the urea cycle. Increased levels of the precursors to neurotransmitters of the brain.
	[36]	Al-Kuraishy (2019)	Prospective, randomized, and open-label study	84	Type 2 diabetes mellitus	300 mg and metformin (1 g)	8 weeks	Improvement of endothelial dysfunction and inflammatory changes in patients with T2DM. Amelioration of metabolic profile.
	[37]	Mazza (2018)	Multicentre, randomized, open-label, post-marketing clinical trial	104	Metabolic syndrome	30 mg and Monacolin K (10 mg)	2 months	Reduction of systolic blood pressure, diastolic blood pressure, total cholesterol, LDLC, triglycerides and serum glucose.
	[38]	Kuhlman (2019)	Randomized controlled trial	35	Treatment with a minimum of 40 mg of simvastatin	400 mg	8 weeks	No improvement of peripheral insulin sensitivity.
	[39]	Yen (2018)	Double-blind, randomized, placebo-controlled trial	50	Type 2 diabetes	100 mg	12 weeks	Increase in antioxidant enzyme activity levels, reduction of HbA1c levels and maintaining of HDL-cholesterol levels.
	[40]	Kawashima (2020)	Single-Center, Randomized Double-Blind Placebo-Controlled Crossover Pilot Study	14	Heart failure with reduced ejection fraction	400 mg	3 months	Improvement in peripheral endothelial function.
	[41]	Dludla (2020)	Systematic review and meta-analysis of randomized controlled trials	650	Diabetes or metabolic syndrome	20–400 mg	From 8 weeks to 6 months	Reduction of total cholesterol and LDL.
	[42]	Sabbatinelli (2020)	Randomized, double-blind, single-center trial	51	Subjects with low-density lipoprotein (LDL) cholesterol levels of 130–200 mg/dL, not taking statins or other lipid lowering treatments, moderate (2.5–6.0%) endothelial dysfunction and no clinical signs of cardiovascular disease	100 or 200 mg	8 weeks	Ameliorated dyslipidemia-related endothelial dysfunction.

## Data Availability

The data generated during this study are included in this article and are available on request from the corresponding author.
